# Outbreak of colistin-resistant organisms at a tertiary hospital in Riyadh, Saudi Arabia, 2016

**DOI:** 10.11604/pamj.2019.34.162.19998

**Published:** 2019-11-26

**Authors:** Zayid Al Mayahi, Shady Kamel, Hala Amer, Mark Beatty

**Affiliations:** 1 Field Epidemiology Training Program, Ministry of Health, Riyadh, Saudi Arabia; 2 Department of Family and Community Medicine, King Saud University, Riyadh, KSA; 3 Infection Control Administration, King Saud Medical City, Ministry of Health, Riyadh, KSA; 4 Department of Community Medicine, National Research Centre, Egypt

**Keywords:** Colistin resistance, multidrug resistant organisms, MDROs, antibiotics, KSA

## Abstract

**Introduction:**

Multidrug-resistant organisms (MDROs) have been a major concern in King Saud Medical City (KSMC) recently. The number of cases with colistin resistance was growing rapidly in the first half of 2016, challenging the infection control practices and mandating a thorough outbreak investigation. The objective of this study was to determine the extent of the outbreak, identify potential risk factors and prevent further increase in the rates of MDROs.

**Methods:**

Reviewing the medical records of the 22 admitted cases with colistin resistance using an abstraction form composed of demographical data, comorbidities, details of current admissions, and procedures. Also, tracking patients' movements in the hospital, reviewing all cultures isolates, and reviewing the surveillance and infection control strategies.

**Results:**

Mean age was 49.71±17.824 (20-79 years), 90.9% were males, 63.6% cases admitted under medical unit. The average duration of stay in the ED was 1.23 day. Over 2/3 had hypertension and diabetes mellitus. Majority of patients staying between 20-40 days in the hospital & the average number of days until developing colistin resistance was 44.18. Resistance was solely related to two organisms that were Acinetobacter baumanni (59.1%) and Klebsiella pneumoniae (40. 9%). Ventilators and folly's catheters were equally (95.5%) used by 21 patients. The most common site of infection was respiratory (41.3%), of which most were sputum samples. Resistance of over 75% is recorded by antibiotics like tazocin, ciprofloxacin, imepenen and oxacillin.

**Conclusion:**

The uncontrolled use of antibiotics, prolonged stay in the Intensive Care Unit (ICU), frequent uses of different devices, are the potential risk factors of developing colistin resistance.

## Introduction

Similar to other regions in the world, anti-microbial resistance (AMR) is a severe and worsening problem on the Arabian Peninsula. The scope of AMR in this region has not been well defined due to the lack of systematic surveillance at the national level and easy accessibility to over-the-counter drugs [1, 2]. The unregulated use of the last resort drugs like colistin without adequate cause has also played a major role. As expected, there has been increased rate of colistin resistance [3-6].

## Methods

**Study area:** King Saud Medical City (KSMC) is a premier health center in the kingdom achieving and maintaining joint commission international accreditation in 2013. It has 1400 beds of which 122 are devoted to intensive care. The emergency department is one of the busiest in the kingdom.

**Screening and isolation procedures:** as per screening protocol at KSMC, active surveillance cultures were applied for all ICU admissions as follows: nasal and wound swabs for MRSA, respiratory secretion for patients with respiratory devices and urine sample for patients with urinary catheter. Being one of the main ICUs in KSA, a lot of transfers from other hospitals were accepted to the ICU as there was no mandatory pre-acceptance screening at their original hospital and microbiological data were frequently missed in their referral documents. Some patients who needed ICU admission may spend 1-2 days in the emergency department (ED) waiting for bed allocation without microbiological screening before getting admitted to ICU. All cultures and sensitivity testing were completed in the KSMC laboratory in accordance with the latest edition of Clinical and Laboratory Standards Institute (M100-Performance Standards for Antimicrobial Susceptibility Testing, 28^th^ edition, Clinical and Laboratory Standards Institute). This laboratory is also the regional reference laboratory. However, due to the absence of electronic medical records, the surveillance by the infection prevention and control unit was challenging because patient medical records were often spread out among different units and departments.

**Outbreak:** during 2015 and 2016, MDROs have been a major concern in King Saud Medical City (KSMC). MDROs had significantly increased in 2015 peaking in August. This dramatic increase continued into 2016. Some organisms also developed resistance against colistin, an antibiotic of “last resort” for multidrug-resistant infections, including carbapenem-resistant *Enterobacteriaceae* (CRE) [7]. In February, the KSMC laboratory began a detailed inventory of MDRO also resistant to colistin. Due to higher than expected prevalence of these organisms an outbreak was declared. In July 2016, Saudi Ministry of Health requested physicians from the Riyadh Field Epidemiology Training Program (FETP) to assist in the investigation. The FETP program is an intensive 2-year assignment that provides health professionals practical field training in conducting outbreak and other epidemiologic investigations under the supervision of national and international experts. KSMC accepted the collaborative assistance in late July 2016 for investigation of MDRO, including colistin resistance, and development of prevention and containment measures for the outbreak. Immediately, strict screening of hospital transfers was initiated with cohorting until resistance status was known.

The FETP team conducted thorough inspections of the intensive care units (ICUs) and isolation wards, especially where the transfers were admitted. This investigation included review hospital isolation policies and procedures and observation of compliance with those procedures. In addition, patient medical records, infection control records, and all other relevant documents of persons infected and potentially infected (transferred but no screening result available) with resistant organisms were thoroughly reviewed and abstracted. For this investigation laboratory results on all cultured bacteria with resistance were sent to hospital infection control for review and abstraction into the newly constructed active surveillance database. Indications for including reports included both diagnostic and active screening surveillance cultures. To facilitate objective analysis a standardized data collection form was developed. Data collected included demographic, clinical, and laboratory data. Also, collected were the movements of the patient through the hospital and procedures the patient required. Tracking of the patient movements facilitated by the existing Tanseeq software, an electronic application developed by the King Saud Medical City (KSMC) Information Technology and Bed Management Administrations. A case was defined as any patient with current admission to King Saud Medical City who had a positive culture with documented colistin resistance.

## Results

On September 5^th^, 2015, the first patient with colistin resistance was reported out by the laboratory. Next, on January 4^th^, 2016, a second case was detected. In response an alert system for colistin resistant organisms was established in February: a total of 83 patients harboring colistin resistant organisms were identified; 31 died and 30 cases were discharged. Majority of expired cases were already labelled as end of life cases based on their clinical conditions that met criteria of Do Not Resuscitate DNR policy. The peak of the outbreak occurred during February, March and April 2016, when majority of cases were reported. Among the reported cases were 22 patients still in the hospital in June and July. Table 1 shows the characteristics of the 22 patients with colistin resistant organisms. Ages ranged from 20 to 79 years, with a mean age (+SD) of 50±19. Most of the patients were Saudi (18 patients; 82%) and 20 (91%) were male. There were 14 (64%) cases admitted under medical service, and 8 (36%) cases under surgical care. Two cases were referred from other hospitals, and the remaining 20 (91%) cases were admitted through emergency department (ED). Almost 9 cases (41%) spent less than 24 hours in ED, 10 cases (46%) completed one full day and three cases stayed two days or more. The average duration of stay in ED was 1.2 day.

**Table 1:** Characteristics of patients with colistin resistant organisms (n=22)

Characteristic	Number (n=22)	%
**Age in years:**		
<40 year	7	31.8
40 – 60	8	36.4
> 60 year	7	31.8
Range	20-79	
Mean+SD	49.71 + 17.824	
**Gender**		
Male	20	90.9
Female	2	9.1
**Healthcare service**		
Medical	14	63.6
Surgical	8	36.4
**Type of admission**		
Through Emergency Department	20	90.9
Direct referral from other hospitals	2	9.1
**Time in emergency department**		
Less than 24 hours	9	40.9
One day	10	45.5
Two days	1	4.5
Three days and more	2	9.0
Mean +SD	1.23 + 2.525	
**History of trauma**		
Yes	5	22.7
No	17	77.3
**Decreased level of consciousness**		
Yes	12	54.5
No	10	45.5

**Figure 1:**
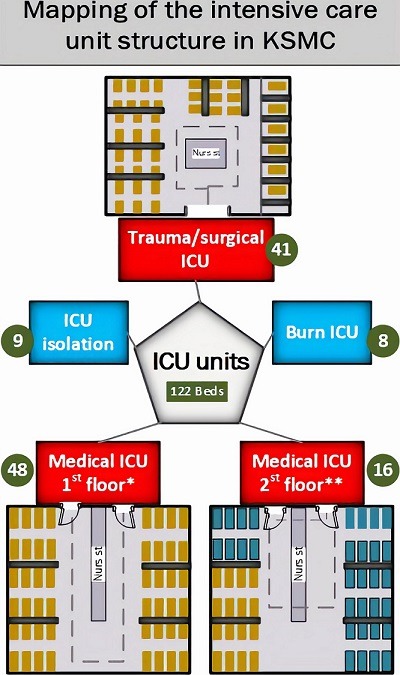
The structure of the intensive care unit

There were 5 patients (23%) who presented with history of trauma (motor vehicle accident and fall from height), and almost 10 patients (46%) presented with decreased level of consciousness. Over two thirds of patients had co-morbidities of hypertension and diabetes mellitus. Patients who also had renal and heart diseases were 18% and 13%, respectively. None of the patients had lung or liver disease or cancers. Only three patients were feverish on admission. The average length of stay (LOS) to develop colistin resistance was 44+54 days. The range was 6 to 271 days. Another finding was the repeated transfer within and between hospital units. On average each patient was moved 4.2 times throughout his/her stay in the hospital. Transfers were mainly (74%) between the specified ICU areas (Trauma/surgical ICU, Medical ICU 1^st^ floor and Medical ICU 2^nd^ floor) and the general medical ward assigned for cohorting of MDROs outside the ICU. In addition, about 72% of MDROs acquisition among the study group occurred within the ICU areas which later progressed to colistin resistance. Figure 1 illustrates the huge ICU structure in KSMC. Medical devices most frequently used for patients who developed colistin resistant organisms were ventilators and Foley catheters (96% each). The most common specimens of positive cultures were respiratory (41%), of which most were sputum samples. Blood infections were second (22%) followed by soft tissue and skin infections (21%) and urinary tract infections (15%). Table 2 illustrates this in more detail.

**Table 2:** Distribution of sites of infection and cultures sampled

Site of infection	Samples			Total Number N=131	Percentage of patients on devices N=22
	Type	Number (131)	%		
Respiratory tract	Sputum	41	31.3	54 (41.3 %)	Ventilator 21 (95.5%)
	Tracheal aspirate	12	9.2		
	Pleural fluid	1	0.8		
	Bronchial wash	1	0.8		
Soft, skin, tissue	Soft, skin & tissue	28	21.4	28 (21.4%)	
Blood stream	Blood	28	21.4	29 (22.1%)	
Urine tract	Urinary catheter	11	8.4	19 (14.5%)	Urinary catheter
	Urine	8	6.1		21 (95.5%)
CSF	CSF	1	0.8	1 (0.8%)	

The total number of positive microbiological cultures for the 22 patients was 131 (6±3 per patient). Of which, 103 (79%) were caused by gram negative organisms. The top four organisms most commonly seen were Klebsiella pneumonae (18%), *Acinetobacter baumanni* (17%), *P. aeruginosa* (13.7%) and *Proteus mirabilis* (9.9%). Cultures which showed resistance to at least one type of antibiotic other than colistin were 108 (82%). There were 31 (24%) cultures classified as multidrug-resistant (MDR). Cultures with Methicillin-resistant *staphylococcus aureus* (MRSA) were 9 (7%), whereas carbapenem-resistant *enterobacteriaceae* (CRE) and extended spectrum beta-lactamases (ESBL) were 15% and 14% respectively. One culture showed Vancomycin resistant *Enterococcus* (VRE). The use of antibiotics in the preceding 72 hours before the positive cultures were recorded in two-thirds of the patients (66%). Table 3 illustrates. Moreover, in regards to 22 cultures which turned positive for colistin resistance, majority (12) were respiratory samples followed by five urinary samples and three blood samples. There were 14 cultures (63.6%) classified as infections, and the rest were colonization. The colistin resistance was solely related to two organisms that were *Acinetobacter baumanni* (59.1%) and *Klebsiella pneumoniae* (40.9%).

**Table 3:** Characteristics of resistant organisms of 131 cultures

Variables	Number (n=131)	%
**Gram stain**		
Gram negative	103	78.6
Gram Positive	28	21.4
Resistance to at least one AB	108	82.4
Multidrug Resistant Organism	31	23.7
MRSA	9	6.9
VRE	1	0.8
CRE	20	15.3
ESBL	18	13.7
**History of Antibiotic use in last 72 hour**		
Yes	87	66.4
No	41	31.3
Unavailable	3	2.3

**Antibiotic resistance:** the antibiotic resistance trends among the 131 studied cultures of the 22 patients show a repeated trend in some antibiotics like colistin. Resistance of over 75% is recorded by antibiotics including piperacillin/tazobactam, ciprofloxacin, imepenen, and oxacillin. Less than 40% antibiotic resistance was recorded by only 5 antibiotics (vancomycin, ampicillin, cefepime, clindamycin, tigecycline), whereas colistin resistance was near to half as Table 4 shows.

**Table 4:** Frequency of antibiotics resistance

Drug	Resistance/Administration	%
Cefotaxime	8 (8)	100
Tazocin	11(13)	84.6
Ceftazidime	39(47)	83
Ceftriaxone	23(28)	82
Ciprofloxacin	75(95)	79
Imipenem	50(64)	78.1
Oxacillin	13(17)	76.5
Nitrofurantoin	8(11)	72.7
Gentamicin	50(79)	63.3
Meropenem	51(82)	62.2
Cotrimox	55(94)	58.5
Amikacin	37(79)	46.8
Colistin	23(53)	43.4
Tigecycline	5(13)	38.5
Clindamycin	4(11)	36.4
Cefepime	12(37)	32.4
(Ampicillin, Amoxicillin)	1(7)	14.3
Vancomycin	1(13)	0.08

**Interventions:** hospital management convened a resistance committee. Upon detection, of MDRO including colistin resistant organisms the patient's record was clearly flagged. Two infectious disease physicians and two clinical pharmacists were instructed to control the “colistin” prescriptions and dosing. In addition, an antibiotic policy was developed to guide health care providers in antibiotic selection and treatment duration to reduce the risk of development of resistance. In response to this outbreak, rectal swab for patients referred from other hospital was added to the routine ICU admission screening procedure mentioned earlier. Moreover, rectal swab was repeated every two weeks for all ICU patients during the entire period of outbreak. As per reviewing infection control process and key performance indicators in ICU, the overall hand hygiene compliance was around 70%. The cases infected and colonized with MDRO were cohorted in dedicated ICU areas where a separate cubic was allocated for each different organism. The contact precautions were followed with all patients in MDROs screening and cohorting areas, and quaternary ammonium-based disinfectants were used for environmental surfaces disinfection 1-2 times per day. Speeding patient discharge to home or returning patients to the facility they came from after the procedure that brought them to KSMC was complete may have been useful additional steps but these were not practicable as families and home care teams declare they were not ready to take care of these types of patients.

## Discussion

Rising antimicrobial resistance is a serious global medical concern. Medications which have broad coverage or have serious side effects (e.g. neurotoxic, nephrotoxic) such as colistin, are currently used as the last resort treatment option for many resistant organisms which are no longer susceptible to other antibiotics [8, 9]. Having extensively reviewed the hospital policies and procedures about antibiotic prescribing, the investigation team concluded the unregulated and frequent use of antibiotics which should be reserved as last resort agents in this hospital may have been the potential cause for this outbreak. The average duration required before developing colistin-resistance was 44 days (6-271). Therefore, all infections with colistin resistance organisms were hospital acquired. There were only two cases which were referred from other hospitals, and got admitted on 29^th^ June and 9^th^ July 2016, their screening swabs shown they already had colistin resistance organisms but in both cases the processing of samples and resistance testing two weeks or more.

The results of the investigation suggest demographic characteristics of the patients do not seem to have any significant role in developing the resistance to colistin (e.g. age and gender). On the other hand, extremely long length of stay in the hospital was common among all patients with colistin resistance. Comorbid health conditions, such as hypertension, diabetes mellitus, renal and heart diseases were common among patients with colistin resistance organisms. However, these conditions may have contributed the need for longer hospital stays. Still patients requiring prolonged instrumentation such as prolonged bladder catheterization and mechanical ventilation. Indeed, all but one of the 22 patients had been ventilated and had an indwelling urinary catheter and the most common type of positive cultures were sputum and urinary samples. Moreover, extended stay in the emergency department for more than 24 hours prior to admission and frequent movements between the different departments and wards in the hospital may contribute to the spread of the resistant organisms to other patients. There are a number of previous investigations of colistin resistant organisms. Results were consistent with the current investigation. Majority of the studies reviewed were agreeable that colistin excessive and uncontrolled use is the most single, crucial and independent risk factor leading to the emergence of colistin resistant organisms [10, 11]. This goes in accordance with the finding of this outbreak investigation. Three previous studies found other risk factors for colistin resistance included: duration and repeated use of colistin, monobactam use, prolonged antifungal use, history surgical procedures or instrumentation including mechanical ventilation, and time spent in an ICU, patient age, history, and comorbidities. However, the multivariate analyses resulted in colistin use as the only significant and independent factor for two studies, whereas the third study found chronic obstructive pulmonary disease (COPD) a significant factor [11-13].

Furthermore, the current investigation indicates the high potentiality of ongoing nosocomial infection in the hospital, though the exact epidemiological linkages need further exploration, and processing with genotyping. Indeed, horizontal transmission with colistin-resistant organisms was also suggested by previous studies [14-17]. The strong indicators are related apparently to the over-stressed infection control practices due to the severe inadequacy of the hospital setup to cope with the fast influx of MDRO cases from inside and outside the hospital. The spread of infection was inevitably accelerated with unreasonable frequent movements of the patients inside the hospital. Patients were mainly identified with colistin resistance while they were admitted in different sections of the ICU, mainly the medical ICU, 1^st^ floor. This also raises the question of a possible continuous source of transmission inside the ICU environment. Staying longer in the ICU reflects the frequent use of antibiotics including colistin, the serious condition of the patients, comorbidities, and frequent uses of devices and procedures. This is consistent with a previous study which concluded that cross transmission is facilitated by frequent use of suctioning to the patient with ventilator associated pneumonia [18]. The exiting fragmented surveillance system in KSMC has also to be enhanced urgently to analyze the situation thoroughly and systematically to produce the effective recommendations. This has already been suggested by a study published recently in 2016 to combat the antimicrobial resistance in Gulf Cooperation Council States, including Kingdom of Saudi Arabia [19]. Enhancement of the surveillance systems should also go in parallel with establishing antimicrobial stewardship programs [17]. This investigation was limited by the lack of a comparison group to conducted multivariate analysis of risk factors and a lack of genotyping capability for isolates. However, the findings were consistent with other published investigations.

## Conclusion

Colistin resistance is an expected result of the over-use of anti-microbials. There are obvious clear factors which played a critical role in augmenting the problem including prolonged hospital stays particularly in ICUs, frequent movements of unclear necessity, comorbidities, unregulated use of antimicrobials, and the need for procedures or instrumentation necessary to support patients. Maintaining a comprehensive nosocomial surveillance system, strict adherence to established infection control procedures and developing antimicrobial stewardship programs would address all of the modifiable risk factors identified.

### What is known about this topic

- Multidrug resistant organisms are among the most serious health problem globally;
- Many causes are hypothesized for this issue, including the excessive and unregulated use of Antibiotics, the inherited ability of the organisms to overcome the antibiotics;
- Experts are convinced on the urgent need to utilize all information from all the world to help make practical recommendations and at least reduce the speed of resistance development.

### What this study adds

- It demonstrates the malpractice in one of the biggest hospitals which are supposed to be the modal for the best practice;
- It supports the global understanding that the increased and unnecessary use of antibiotics is the main key behind this serious problem;
- It also demonstrates that the increased and unnecessary stay and movements in the hospital is another factor facilitating the nosocomial transmission in the hospital.

## Competing interests

The authors declare no competing interests.
